# Infant Neural Sensitivity to Dynamic Eye Gaze Relates to Quality of Parent–Infant Interaction at 7-Months in Infants at Risk for Autism

**DOI:** 10.1007/s10803-014-2192-9

**Published:** 2014-07-30

**Authors:** Mayada Elsabbagh, Ruth Bruno, Ming Wai Wan, Tony Charman, Mark H. Johnson, Jonathan Green, The BASIS Team

**Affiliations:** 1 Department of Psychiatry, Faculty of Medicine, McGill University, 1033 Pine Avenue West, Montreal, QC H3A 1A1 Canada; 2 Centre for Brain and Cognitive Development, Birkbeck, University of London, London, UK; 3 Institute of Brain, Behaviour and Mental Health, University of Manchester, Manchester, UK; 4 Manchester Academic Health Sciences Centre, University of Manchester, Manchester, UK; 5 Department of Psychology, Institute of Psychiatry, King’s College London, London, UK

**Keywords:** Infant, Autism, Interaction, EEG, Familial risk, Prospective study

## Abstract

Links between brain function measures and quality of parent–child interactions within the early developmental period have been investigated in typical and atypical development. We examined such links in a group of 104 infants with and without a family history for autism in the first year of life. Our findings suggest robust associations between event related potential responses to eye gaze and observed parent–infant interaction measures. In both groups, infants with more positive affect exhibit stronger differentiation to gaze stimuli. This association was observed with the earlier P100 waveform component in the control group but with the later P400 component in infants at-risk. These exploratory findings are critical in paving the way for a better understanding of how infant laboratory measures may relate to overt behavior and how both can be combined in the context of predicting risk or clinical diagnosis in toddlerhood.

**Electronic supplementary material:**

The online version of this article (doi:10.1007/s10803-014-2192-9) contains supplementary material, which is available to authorized users.

## Introduction

In recent years, several studies on autism have investigated possible early behavioural markers of the disorder. As yet, a behavioural diagnosis is rarely made before the child reaches the age of 2–3 years (Charman and Baird 2002; Zwaigenbaum et al. 2009). A large body of evidence from studies of infants at-risk (by virtue of having an older diagnosed sibling) suggests that overt social and communicative behaviours may appear typical in this group within the first year of life, but signs of autism begin to emerge at around 12 months of age in some infants (e.g.Ozonoff et al. 2010; Zwaigenbaum et al. 2005). In contrast, within the first year brain function measures have identified differences in infants at-risk relative to infants with no family history of autism (e.g. Elsabbagh et al. 2009, 2012; McCleery et al. 2009). A recent study suggested that some of these early brain function differences are associated with a later autism diagnosis (Elsabbagh et al. 2012). Nevertheless, we still have a limited understanding of this seeming discrepancy in the timing of detection of neural and behavioural manifestations of risk in infants with a family history of autism. It has been suggested that early subtle differences in brain function within the first year may become compounded and amplified due to atypical interactions within developing brain systems and with the external environment, leading to the emergence of atypical behaviours in the second year in some individuals (Elsabbagh and Johnson 2010).

Such dynamic and complex developmental pathways are difficult to test empirically. However, transactional models of parent–child interaction (PCI) (e.g. Sameroff 2009) have also raised the possibility that even very subtle manifestations of risk may disturb natural mutuality in social interactions (Wan et al. 2012, 2013). These models suggest that systematic observation of early PCI can be used to identify the presence of early social interaction atypicalities that influence later development, potentially including a diagnosis of autism. From birth onwards, parent and child enter into a dyadic relationship of mutual influence that shapes their interaction over time and sets the foundation for future social relationships. Thus, early disruptions in the child’s social communication skills may affect the parent’s pattern of response, which in turn may exacerbate early difficulties with social competence and lead to an increasingly atypical developmental trajectory (Dawson 2008; Elsabbagh and Johnson 2007, 2010). While it has been extensively shown that autism is not the result of poor parenting, parental and infant response in an interactional setting may on the one hand contribute to an atypical social developmental trajectory and, on the other, be useful as a sensitive detector of early social atypicalities on the child’s part.

Evidence for such disruption in the interaction between children diagnosed with autism and their caregivers has been presented in previous studies. On the one hand, parents of diagnosed children show levels of responsiveness or synchrony similar to those of caregivers of children with developmental delay and typically developing children (Siller and Sigman 2002). However, parents of children with autism tend to use a more directive style of interaction because their children respond less to social cues than a typically developing child (Doussard-Roosevelt et al. 2003; el-Ghoroury and Romanczyk 1999; Lemanek et al. 1993). While the parent would use this directive style in an attempt to further engage the child, studies suggest that a directive interaction style may effectively cause some children to disengage from the parent (Lussier et al. 1994) and may contribute to lower attentiveness to caregiver in play interactions (Sigman et al. 1986). Consistent with this pattern, a retrospective study examining PCI prior to the onset of overt autism symptoms, found that that relative to typically developing infants, parents of children who later develop ASD tend to be more intrusive during the first year of life and their infants are more likely to withdraw during the second year (Saint-Georges et al. 2011).

Taken together, these findings suggest that in autism, initial atypicality in developing brain systems may relate to a reduced level of infant synchrony with their caregiver and increased infant disengagement, which in turn contribute to a less than optimal environment for social development. Recently, prospective longitudinal studies have begun to systematically test these theoretical proposals in infants at-risk for autism by virtue of having an older diagnosed sibling. The first studies in this area have examined potential differences in early parent–infant interaction between dyads of siblings at-risk of autism relative to control groups of dyads with no family history of autism. For example, Yirmiya et al. (2006) microanalytic study of 4-month-old at-risk siblings found that a significant proportion had less synchronous interactions when infants led play compared to low-risk groups. These findings suggest that there may be subtle early atypicalities in infant-initiated affect within social interaction. In contrast, a study of 6-month-old at-risk infants in face-to-face free play interaction found that the ASD group at 6 months (*N* = 8) did not differ from at-risk infants who do not develop ASD and low-risk comparisons in the frequency of gaze, smiles, and vocalizations directed toward the caregiver, nor in their sensitivity to her withdrawal from interaction (Rozga et al. 2011). However, the lack of differences in this sample is not surprising in view of the small sample size. Another study found that global ratings of parent nondirectiveness and sensitive responsiveness, and infant liveliness, during free play interaction at 7 months differentiated at-risk infants from the low-risk group (Wan et al. 2012). Although parent–infant interaction characteristics in these areas at 7 months did not predict later autism outcome (Wan et al. 2013), any emerging atypicalities in social interaction may manifest themselves in more subtle ways in early infancy.

Despite offering some insights into the nature of PCI in the development of autism, these findings are still limited as far as addressing the questions raised by transactional or developmental models. Specifically, during the first year, brain function measures appear to be more sensitive in signaling manifestations of risk that are associated with diagnosis in toddlerhood (Elsabbagh et al. 2012; Elsabbagh and Johnson 2010). Nevertheless, links between such brain function measures and quality of parent–child within the early developmental period have never been directly addressed in the context of autism. Understanding such links between these may prove useful in identifying whether PCI can have a mediating influence on developmental trajectories. For example, a number of studies with infants of depressed mothers have indicated that infants as young as 3–6 months of age show greater relative right frontal asymmetry compared to controls, as well fewer instances and a lower intensity of empathic behaviours and more reported behaviour problems (Field et al. 1995; Jones et al. 1997, 2000).This pattern was observed both at baseline and during a variety of social interactions with mothers and strangers, and was still present at 13–15 months of age (Dawson et al. 1999). Follow-up studies (Diego et al. 2002, 2006) drew a further distinction between depressed mothers with either an intrusive or a withdrawn parenting style, and observed that compared to children of intrusive depressed mothers and controls, children of withdrawn depressed mothers showed greater relative right frontal asymmetry. Fox et al. (1995) observed that higher left frontal EEG resting activity was predictive of higher later social competence. Inversely, greater right frontal asymmetry, as was observed in infants and children of depressed mothers, was predictive of lower social competence at 4 years of age.

Similar links between behavioural and electrophysiological responses have been reported in typical development. Event related potential (ERP) components of interest include the P400—a positive ERP component related to processing of faces known to be atypical in those diagnosed with autism—and the Nc, a negative ERP component related to attention and recognition memory. For example, infants with highly positive mothers showed a larger Nc component to fearful than happy faces (de Haan et al. 2004). Further, Swingler et al. (2010) investigated a group of typically developing 6 month-olds on measures of distress and visual search following a brief period of maternal separation. Results indicated that high levels of distress were associated with a larger amplitude of the P400 and the Nc components when shown images of the mother’s face. Visual search in response to maternal separation was associated with longer P400 and Nc latencies in response to images of a stranger’s face.

These findings from typical and atypical development highlight that our understanding of the developmental pathway leading to autism may be informed by a clearer view of the relationship between brain function measures and PCI. We investigated this question in a cohort of 104 infants followed from the age of 6–10 months to 36 months. Previous reports in two separate studies on the same cohort reported that infants at-risk for autism are distinguishable in ERP responses to face and gaze stimuli presented on a screen, as well as in patterns of play interaction with their caregivers in an unstructured environment within the first year of life. Electrophysiological responses to viewing dynamic gaze shift (i.e. differential response to gaze facing towards and away from the infant) were recorded as previously described in detail (Elsabbagh et al. 2012). Our previous findings suggested that characteristics of gaze-sensitive ERP components P100 and the P400, but not the N290 distinguished infants at-risk from the control group. Infants in the control group exhibited significantly shorter P100 latency and lower P400 amplitude for faces looking towards versus faces looking away from them whereas infants at-risk did not reliably distinguish the two stimuli. When examined against diagnostic outcomes at 3 years, we found that characteristics of the P400 distinguished those infants who later received an ASD diagnosis.

In the PCI assessment, a 6-min episode of unstructured play was videotaped during which the parent–infant dyad was seated on a floor mat in a room with a small set of toys. The parent was instructed to engage in play with their child as they would do at home with or without the use of toys. Our findings suggest that at 7-months, the interactions in at-risk sibs were marked by lower infant liveliness, and reduced parent sensitive responsiveness and nondirectiveness (Wan et al. 2012). These data, which were collected concurrently with the ERP task, were considered for the current study. However, it is worth noting that our previous work indicated that differences in interaction became amplified by 13-months to affect dyadic mutuality and engagement intensity, infant attentiveness, infant affect and parent nondirectivness (Wan et al. 2013). In addition, dyadic mutuality, infant affect and infant attentiveness at 13-months were associated with a later ASD diagnosis at 3 years of age.

### Overview of the Current Study

We tested whether ERP differences observed in groups of infants at high and low risk for autism were associated with measures of PCI collected concurrently at the age of 7-months. At one extreme, we might expect laboratory measures to be unrelated to those observed within the less structured context of free play interaction. On the one hand, ERPs measure the infant brain’s direct response to very specific social stimuli features, i.e., gaze contrast in a highly structured laboratory setting. In contrast, PCI observational measures focus on infant’s overt behaviour within a dyadic context of free play with a caregiver. Moreover, as suggested earlier, brain function measures across a range of previous studies have often distinguished infants at-risk as a group from controls within the first year of life, whereas overt behavioural or social interaction measures tend to distinguish these groups only later in the second year (Elsabbagh and Johnson 2010). However brain function measures including ERPs may be capturing subtle and early emerging differences that become more evident over time at the behavioural level. The latter view predicts that within the first year measures of naturalistic interactions should be associated to some extent with brain function measures, signaling an increasingly atypical developmental trajectory, the effects of which are only observed more clearly in the second year of life. Our goal in the current study was to test this possibility by examining whether laboratory ERP measures to socially relevant stimuli are associated with measures of PCI within the first year of life. Of interest was the extent to which any patterns of association were similar or different in infants at-risk relative to the control group. Finally, given the novelty of our approach, we also include consideration of the methodological and analytic challenges in mapping neurophysiological onto PCI measures as well as future directions to address them.

## Methods

### Sample

The original sample of participants included 104 families recruited sequentially into the study. Within this group, there were 54 at-risk infants (21 male, 33 female) and 50 control infants (21 male, 29 female). The infants were on average 7-months at the time of the study (mean = 238.3 days, SD = 37.2). Of 104 dyads recruited, data from 12 were excluded from the current study because interaction tapes were not collected (n = 4) or due to technical problems (no clip or too brief: n = 3; no sound: n = 5). The final sample comprised 45 at-risk infants [20 male, 44.4 %; mean age = 7.16 months (SD = 1.2)] and 47 comparison low-risk infants [18 male; 47.4 %; mean age = 7.36 months (SD = 1.2)]. All parents who took part in the interaction were mothers. The study had received ethical approved by London National Health Service Research Ethical Committee and informed consent was obtained from one or both parents. At the time of the study, none of the infants had been diagnosed with a developmental disorder or other medical condition. At-risk status was defined as having an older sibling (in 4 cases, a half-sibling) with a community clinical diagnosis of ASD. Clinical diagnosis of the older sibling was confirmed by two expert clinicians based on the Development and Wellbeing Assessment (DAWBA; Goodman et al. 2000) and the parent-reported Social Communication Questionnaire (SCQ; Rutter et al. 2003). Most of the older siblings met criteria on the DAWBA and SCQ. Low-risk infants were recruited separately from volunteers at the Centre for Brain and Cognitive Development. All low-risk infants had at least one older sibling or half-sibling screened for possible ASD using the SCQ, with none scoring above threshold (one score was missing).

Data from two tasks, previously used with this cohort were selected for the current study In the ERP task, infants were seated on their parents’ lap and facing towards a computer screen on which were images of female faces with gaze directed either towards or away from the infant. Each trial block began with a static colourful fixation stimulus followed by three to six gaze shifts by the presented face, alternating from directing gaze towards or away from the infant. Brain activity was recorded using 128-channel hydrocel nets. Segmented data was processed using standard procedures including baseline correction, manual artefact rejection, interpolation of missing channels, referencing to the average. Three task-sensitive components (P100: 120–199 ms, N290: 200–319 ms, and P400: 320–520 ms) were ascertained over the occipito-temporal region, and their characteristic amplitude and latency were computed for each infant. Further methodological details on the set up, channel selection, and signal processing have been previously reported (Elsabbagh et al. 2012).

In the PCI assessment, a 6-min episode of unstructured play was videotaped during which the parent–infant dyad was seated on a floor mat in a room with a small set of toys. The parent was instructed to engage in play with their child as they would do at home with or without the use of toys. After a brief period of familiarisation, the researcher left the room—this was the point from which the video clip is later independently evaluated, blind to all participant information.

A global rating scheme, the Manchester Assessment of Caregiver-Infant Interaction (MACI; Wan et al. 2012, 2013), was developed for the wider project and used to evaluate interactions by rating seven items on a 1–7 scale: Caregiver sensitive responsiveness (the degree of appropriate and contingent behavioural responding to identified infant behaviours that support the infant’s interaction and development), caregiver nondirectiveness (the degree of behavioural and verbal focus on the infant’s experience and agenda as opposed to a caregiver-directed focus such as demands and intrusions), infant attentiveness to caregiver (the amount and quality of visual contact with an interest in the caregiver directly or through mutual focus), infant positive affect (the amount and extent of positive mood that includes positive expression and vocalisation, weighed against negative affect and behaviour), infant liveliness (the level of voluntary physical activity, particularly that initiated by the infant), dyadic mutuality (the degree of dyadic sharedness and reciprocity of experience) and dyadic intensity of engagement (the intensity, not quantity, of mutual engagement at its most optimal). From the original sample, data on both gaze ERP measures and PCI measures were available for 84 children (43 control and 41 at-risk).

### Data Analysis

The ERP waveform contained 12 potential variables (amplitudes and latencies for each component for each condition). PCI measures contained seven 7-point Likert-scales. Given the large number of measures derived from the ERP and PCI task, and the fact, two analytic steps were taken to reduce the number of variables. First, response difference was used to the target stimulus contrast, rather than absolute response to each condition. Second, principal component analysis (PCA) on standardized measures (because latency and amplitude variables have different ranges) was used to reduce variables by quantifying the number of independent variables (orthogonal directions) accounting for at least 80 % of variance. In relation to the factor structure of PCI measures, exploratory factor analysis was performed in the previous study (Wan et al. 2012) suggested that with eigenvalue >1.0, two factors accounted for most of the variance. Only remaining variables were used to test possible association between ERP and PCI. (See supplementary materials for details). Univariate and bivariate analyses were performed to evaluate some assumptions regarding the distribution of the selected variables. All measures were screened for outliers and Shapiro–Wilk tests were use to assume distribution’s normality. Shapiro–Wilk tests confirmed that ERP latency measures were normally distributed within each group (*p* values ranged from 0.15 to 0.21).

Univariate and bivariate analyses were performed to evaluate some assumptions regarding the distribution of the dependents variables and to identify potential PCI variables that predict ERP variables. All measures were screened for outliers and Shapiro–Wilk test were use to assume distribution’s normality. Pearson Correlation and linear regression models were performed by group, using the same approach, to determine correlation coefficient on each group. Stepwise selection model was used specifying a *p* value <0.10 for entering in the model, and a *p* value <0.05 for stay in the model. R^2^, AIC (Akaike Information criterion) and BIC (Bayesian Information criterion) criterion were used for select the best model. Model validation was examined using collinearity diagnostic tools [variance inflation factors (VIF)], and homocedasticity test of residuals variance.

## Results

Based on the reduction process detailed in the supplementary materials, the following were used for subsequent analysis: P400 latency and amplitude and P100 latency as ERP predictors, as well as infant positive effect, parent attentiveness, infant liveliness, parent sensitive responsiveness, and mutuality as PCI dependent variables. Three stepwise regression models by group were performed of the ERP measures.

In the first regression model, a significant main effect of parent sensitive responsiveness (F = 8.9, R^2^ = 0.18, *p* = 0.004) was observed on the P100 latency difference in the control group. No significant effect were observed in the at-risk group.

In the second regression model, none of the PCI independent variables were significantly associated with P400 amplitude difference in either group. Uncorrelated P400 amplitude difference coefficient ranged from 0.10 to 0.20 in the control group, and from −0.19 to 0.08 in the at-risk group. Therefore, despite a significant difference in P400 amplitude difference between groups (control vs. at risk) (*p* = 0.01), this measure showed no association with PCI.

**Fig. 1:**
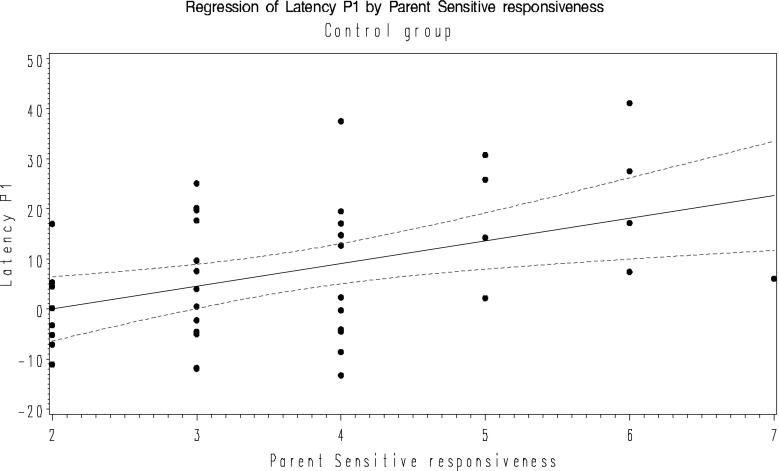
Regression of P100 latency and parental sensitive responsiveness in the control group with 95 % CI. Latency P1 = −9.03 + 4.52 parent sensitive responsiveness

**Fig. 2:**
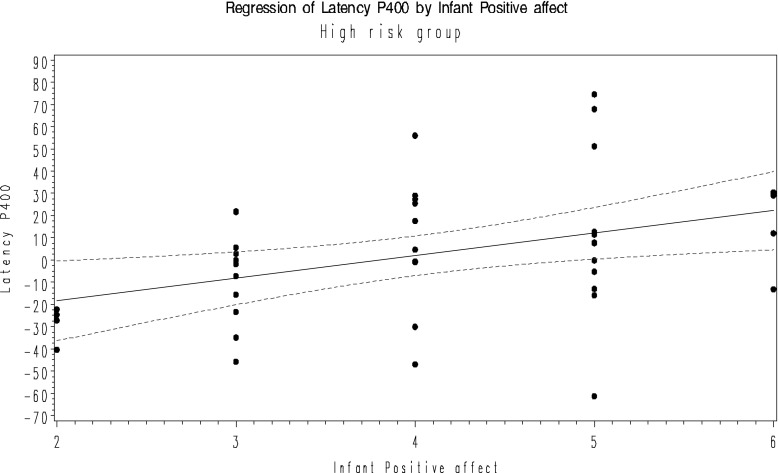
Regression of P400 latency and infant positive affect in the at-risk group with 95 % CI. Latency P400 = −38.7 + 10.2 infant positive affect

In the final model, P400 latency difference was significantly associated with infant positive affect and mutuality in the at-risk group. After testing for multicolinearity (VIF) near to 2 (1.86), correlation coefficient between infant positive affect and mutuality (r = 0.56, *p* < 0.0001), we omitted mutuality (coefficient is closest to zero) and retained infant positive affect in the model (F = 7.1, R^2^ = 0.15, *p* = 0.01). No significant effects were observed in the control group. Figures 1 and 2 show the relationship between ERP and PCI measures in the control and at-risk groups respectively.

Based on the observed patterns of association, we conducted planned follow-up testing to account for potential outcome effects. Here, we focused on the association observed within the at-risk group between P400 latency and infant positive affect. We explored whether the association is specifically driven by infants who were later diagnosed with ASD (n = 13). We ran a Pearson correlation analysis within each of these groups based on their outcome. Within the at-risk group, the observed association between P400 latency difference and infant positive affect was primarily driven by infants who do not develop ASD (F = 10.1, R^2^ = 0.3, *p* = 0.004) rather than those who go on to a diagnosis in toddlerhood (F = 0.2, R^2^ = 0.02, *p* = 0.7).

## Discussion

This preliminary analysis suggests associations between infant brain function measures and particular aspects of observed parent–infant play interaction within the first year of life in infants at-risk of ASD. While associations between the two tasks were found in both high and low risk groups of infants at 7-months of age, the pattern of association differed according to risk status. Our key findings suggest that infants with more positive affect in the at-risk group affect exhibit clearer brain P400 response differentiation to gaze stimuli in the expected direction, i.e., faster response to faces looking toward versus away from the infant.

Specifically, a positive affective state within the context of PCI appears to be tightly linked to development of specific functional brain systems in infants at-risk. While the same direction of association was observed in the control group, infant positive affect was not as strongly associated with brain function measures in this group. In contrast, higher parental sensitivity in the context of interaction in the low-risk control group was associated with greater P100 differentiation of gaze toward versus away from the infant, a pattern not observed in the at-risk group.

A number of factors may underlie the observed difference in the relationship between PCI measures and the specific waveform response component to gaze stimuli. One possibility is that the association in the at-risk group is driven by atypical developmental processes in the subgroup that develop ASD as toddlers. However, our analyses suggest that the association between ERP and PCI measures is not driven by infants who later go on to an ASD diagnosis. In this small sample of infants, these findings may suggest that decoupling of these developing systems in infants who develop ASD. This pattern may also be accounted for by the fact that infants who go on to develop autism show little differentiation between gaze towards versus away from them, reducing the likelihood that a systematic association with interaction measures can be observed (Elsabbagh et al. 2012). However, the power to detect correlational effects in the ASD group is diminished due to the sample size and therefore replication with larger sample sizes is needed. An alternative approach to address this issue in larger samples is to test the hypothesis that combinations of risk factors in infancy, such as those indexed by the ERP and PCI, are associated with severity of symptom expression (Elsabbagh and Johnson 2010).

Another possibility is that differences in patterns of association between ERP and PCI measures reflect diverging developmental pathways underlying brain development in infants at-risk relative to the control group. Specifically, the P100 is known to be responsive to early visual processing to face and gaze information whereas the P400 reflects top-down modulation of visual information and integration across different brain systems (Elsabbagh et al. 2012). As such, infants in the at-risk group may engage qualitatively different mechanisms in interpreting the social value of gaze information. These alternative neural processing pathways relate to the same overt behaviour, i.e. positive affect, in the context of play interaction. Improved understanding of how different neural pathways map onto the same behavioural response in the context of interaction with others may offer important clues into potential protective factors supporting the development of brain networks underlying social cognition in infants at risk who do *not* go on to a diagnosis (Elsabbagh et al. 2012; Kaiser et al. 2010).

In view of these findings, what is the nature of the observed links between ERP response and measures of PCI? It has been suggested that the typically developing infant brain needs to be actively recruited into the social world over the early months and years (Johnson In press). This recruitment process likely requires both the invitation to interact from surrounding adult humans, and an appropriate response from the infant’s developing brain. Previous work shows that aspects of brain function in infants are heavily shaped by the quality and nature of the infants’ interactions with their caregivers (de Haan et al. 2004). While both risk groups in the present study showed an association between parent-child interaction and one component of the ERP waveform, it was only in the control group that we observed an association between parental sensitive responsiveness and P100 latency, an early component in the waveform response that is associated with visual analysis of face and gaze information. As such, infant neural functioning in the control group may be differentially influenced by parental interaction patterns relative to the at-risk group. A speculative interpretation of the difference in the pattern of association is that the brains of infants in the control group may be more likely to accept the parental invitation to be ‘tuned up’ appropriately in order to acquire the skills for the complexities of the social world. Further implications of this view may be worthy of future investigation. For example, is there a sensitive period within which caregivers can influence the brain development of their infant, and do they spontaneously withdraw their attempts to interact beyond this time?

The association between processing gaze information and interaction is consistent with what one might theoretically expect within the interactional dynamics discussed in the Introduction. Across both risk groups, variation in ERP response difference may reflect delay or perturbation in how infants process social information contained in the direct versus the averted gaze. Such delay in the infant may be detected by their social partners, even subtly as a lack of predictability (in both speed and nature of response) or regularity or fluency in the infants’ response to the mutual gaze aspects of interaction. The issue of predictability may be key. Rapid, real-time, fluent social interaction is made possible by subtle anticipation of the other’s future behaviours and responses in order that one may time one’s own behaviour in response. This may happen at the simple behavioural level in making generalisations ahead from previous behaviour, but it may also happen at the level of anticipating intent. Better understanding and anticipation of the intent of others (i.e., the underlying organisation of behaviour in relation to current state of mind or longer-term traits or relationships) should allow a deeper level of behavioural prediction and thus maximal fluency of interaction. In this sense it is possible that gaze-processing difficulties in both at-risk and control infants could perturb this fine-tuned mutually goal-directed structure of interaction. We might then expect to see a falling off in fluency or timing in adult responses and a disruption to synchrony and dyadic mutuality in the interaction. This is because a lack of predictability would impede the attunement of the play experience. Supporting this idea, we recently reported stability of dyadic mutuality between 7- and 13-months of age in the control group but not in the at-risk group, with infants later diagnosed with ASD showing significantly less mutuality at 13 months that the rest of the group (Wan et al. 2013).

These findings are exploratory but suggest important first steps towards a better understanding of how infant laboratory measures relate to observation of behaviour, and how both can be combined in predicting risk or clinical diagnosis in toddlerhood. Gaining further confidence in the current findings and extending them to other measures will require addressing a number of methodological challenges in future studies. First, despite the current study being among the largest to employ brain-imaging methods with infants at-risk, the sample size was still insufficient to test the proposed hypotheses within the diagnosed group. Data pooling across samples collected from multiple laboratories will be required, both for replication of the current findings, as well as allowing for the same hypotheses to be tested in the subgroup of infants at-risk who are later diagnosed with autism.

Finally, the observed associations do not necessarily implicate causal mechanisms between processes measured directly in the infant brain and those measured in the context of unstructured interactions. Moreover, our review of the literature in the Introduction suggests that even in typical development such links between specific ERP components and the infant’s behaviour in the broader developmental context are yet to be more clearly defined. The findings of association between infant ERP variation and real-time social interaction are preliminary, but they provide an important new indication of how different levels of brain and behavioural functioning may be related. The dissection of such associations will be a key underpinning to a more fine-grained understanding of the dynamics of early social interaction and development. They provide one testable component of a possible risk pathway between early intrinsic brain processing impairments in infants and later developmental social impairments. Such causal relationships between infant visual processing, interaction, and later social impairments are difficult to test, but interventions which may change one component whilst studying downstream effects on others can provide some evidence of causal association. Interventions that target the modification of parent–infant social interaction may provide the basis of such developmental test (see Dawson et al. 2012; Green et al. 2013).

## Electronic supplementary material

Below is the link to the electronic supplementary material.

Supplementary material 1 (DOC 125 kb)
