# Supplementary material for: Infant Neural Sensitivity to Dynamic Eye Gaze Relates to Quality of Parent–Infant Interaction at 7-Months in Infants at Risk for Autism
Source: J Autism Dev Disord. 2014 Jul 30;45(2):283–91. doi: 10.1007/s10803-014-2192-9 (PMC4309912; doi:10.1007/s10803-014-2192-9)
Supplement: Supplementary file 1 — Supplementary material 1 (DOC 125 kb) [file 10803_2014_2192_MOESM1_ESM.doc]

**INFANT NEURAL SENSITIVITY TO DYNAMIC EYE GAZE IN RELATION TO QUALITY OF PARENT-INFANT INTERACTION IN INFANTS AT RISK FOR AUTISM**

*Supplementary Materials: Data structure and selection of variables*

Given the large number of theoretically independent variables derived from ERP and PCI tasks, a number of steps were taken to reduce these variables, using theory- and data-driven considerations. The first data reduction measure was to compute amplitude and latency response difference between the two stimulus conditions (gaze toward vs. away) for the three ERP components. There are a number of reasons for using the response difference than using absolute amplitude and latency measures for each condition independently: (1) this is a more conservative approach because ERPs are conventionally interpretable as difference scores between the two stimulus conditions being compared, (2) the response difference is susceptible to less potential confounds (for details, see (Reference Blinded)), and (3) this method reduces the number of associations to be considered because the stimulus contrast, e.g., gaze toward vs. away can be considered as a single variable for the association rather than response to each condition separately.

In addition to using the difference measure, Principal component analysis (PCA) were used to ascertain the factor structure within the ERP measures and to identify possible correlations among measures within each task. PCA is a dimensional reduction technique, which identifies orthogonal directions of maximum variance in the original data. Factor structure and correlation among ERP measures is presented in Tables A1 to 3. PCA on standardized ERP amplitude and latency measures for the whole sample demonstrated that the three first principal components are accounted for 84% of the total variance, with amplitude measures for the three components accounting for for 44.4%, P100 and N290 latency for 21.4 %, and P400 latency for 17.7% (Table A1).

Note that all Amplitude ERP measures are positively intercorrelated (Table A2; r=86% to 96%) as well as correlated with the first component (Table A3; r=68% to 85%). Therefore, the first component captured a high degree of redundancy of amplitude measures. Moreover, all amplitude measures made approximately equal contributions to the PCA factor (Table A2). Similarly, P100 and N290 latency were correlated with each other (Table 2; r= 27%) and with the second PCA factor (Table A3; r=72% to 83%). The two measures did not differ in their contribution to the second factor (Table A2). Finally, P400 latency was the only factor correlated with the third PCA factor.

**Table A1-1: Factor analysis Eigen values for ERP measures(Whole sample)**

| Factor | Eigenvalues | Proportion | Cumulative |
| --- | --- | --- | --- |
| C1 | 2.663 | 0.444 | 0.444 |
| C2 | 1.284 | 0.214 | 0.658 |
| C3 | 1.063 | 0.177 | 0.835 |
| C4 | 0.735 | 0.123 | 0.958 |
| C5 | 0.165 | 0.028 | 0.985 |
| C6 | 0.089 | 0.015 | 1.0 |

**Table A1-2: Factor analysis Eigen values for ERP measures (Control vs at risk group)**

|  | Control Group | |  |  | At Risk Group | |
| --- | --- | --- | --- | --- | --- | --- |
| Factor | Eigenvalues | Proportion | Cumulative | Eigenvalues | Proportion | Cumulative |
| C1 | 2,511 | 0,419 | 0,419 | 2,770 | 0,462 | 0,462 |
| C2 | 1,472 | 0,245 | 0,664 | 1,116 | 0,186 | 0,648 |
| C3 | 1,107 | 0,185 | 0,848 | 1,014 | 0,169 | 0,817 |
| C4 | 0,589 | 0,098 | 0,946 | 0,917 | 0,153 | 0,970 |
| C5 | 0,233 | 0,039 | 0,985 | 0,107 | 0,018 | 0,987 |
| C6 | 0,089 | 0,015 | 1,0 | 0,076 | 0,013 | 1,0 |

**Table A2: ERP measures correlation and contribution to each PCA factor (PC: Principal component; CTR= % contribution)**

|  |  | PC1 | PC2 | PC3 | CTR1 % | CTR2 % | CTR3 % |  |
| --- | --- | --- | --- | --- | --- | --- | --- | --- |
| **Amplitude** | | | | |  |  |  |  |
| P100 | Correlation | **0.86** | 0.04 | 0.28 | 27.6 | 0.12 | 7.21 |  |
|  | P | **<.0001** | 0.7 | 0.01 |  |  |  |  |
| N290 | Correlation | **0.96** | 0.05 | 0.13 | 34.57 | 0.19 | 1.52 |  |
|  | P | **<.0001** | 0.6 | 0.2 |  |  |  |  |
| P400 | Correlation | **0.93** | 0.09 | -0.22 | 32.38 | 0.60 | 4.61 |  |
|  | P | **<.0001** | 0.4 | 0.031 |  |  |  |  |
| **Latency** | | | | |  |  |  |  |
| P100 | Correlation | 0.32 | -0.72 | 0.12 | 3.7 | 40.4 | 1.33 |  |
|  | P | 0.002 | **<.0001** | 0.25 |  |  |  |  |
| N290 | Correlation | 0.13 | **0.83** | -0.18 | 0.64 | 53.61 | 3.11 |  |
|  | P | 0.2 | **<.0001** | 0.08 |  |  |  |  |
| P400 | Correlation | -0.18 | 0.26 | **0.94** | 1.19 | 5.09 | 82.2 |  |
|  | P | 0.08 | 0.012 | **<.0001** |  |  |  |  |

**Table A3. Pearson correlation between ERP measurement** (r and p-value)

|  |  |  |  | Amplitude |  | Latency |  |
| --- | --- | --- | --- | --- | --- | --- | --- |
|  |  |  | N290 | P400 | P1 | N290 | P400 |
|  | P1 | Correlation | 0.81 | 0.68 | 0.097 | -0.04 | 0.05 |
|  |  | P | <.0001 | <.0001 | 0.35 | 0.71 | 0.63 |
| Amplitude | N290 | Correlation |  | 0.85 | 0.29 | 0.14845 | -0.03751 |
|  |  | P |  | <.0001 | 0.005 | 0.15 | 0.72 |
|  | P400 | Correlation |  |  | 0.20 | 0.22 | -0.32 |
|  |  | P |  |  | 0.052 | 0.033 | 0.002 |
|  | P1 | Correlation |  |  |  | -0.27 | -0.06 |
| Latency |  | P |  |  |  | 0.008 | 0.54 |
|  | N290 | Correlation |  |  |  |  | 0.07 |
|  |  | P |  |  |  |  | 0.53 |

Based on these findings, and in view of our previously reported results indicating that the P100 and P400 are the two components showing more task sensitivity relative to the N290, (reference blinded), P100 latency and P400 amplitude and latency were retained for subsequent analysis.

In relation to the factor structure of PCI measures, exploratory factor analysis performed on the cohort in a previous study (Reference blinded) suggested that with eigenvalue > 1.0, two factors were accounted for most of the variance. In factor1 infant and dyadic items were loaded, and in factor2 parents items were loaded. Within the first factor, dyadic items (mutuality and intensity of engagement) were highly correlated (r=78%, p <.0001). In contrast, infant items were more modestly correlated (r <50%). Within the second PCA factor, parental sensitivity was strongly correlated with non-directiveness (r = 72%, p <.0001). Infant liveliness did not load strongly onto any of the two factors. Therefore, parental non-directiveness and intensity of engagement were dropped from subsequent analysis, retaining infant positive effect, attentiveness, and liveliness, parent sensitive responsiveness, and mutuality of engagement.

**Table 4. PCI measures : Pearson correlation**

|  |  | Infant attentiveness to parent | Parent sensitive responsiveness | Parent non-directiveness | Infant liveliness | Mutuality | Intensity of engagement |
| --- | --- | --- | --- | --- | --- | --- | --- |
| Infant positive affect | Correlation | 0.49 | 0.43 | 0.26 | 0.30 | 0.56 | 0.55 |
|  | P | <.0001 | <.0001 | 0.01 | 0.005 | <.0001 | <.0001 |
| Infant attentiveness to parent | Correlation |  | 0.56 | 0.25 | 0.14 | 0.85 | 0.73 |
|  | P |  | <.0001 | 0.014 | 0.20 | <.0001 | <.0001 |
| Parent sensitive responsiveness | Correlation |  |  | 0.72 | 0.20 | 0.76 | 0.49 |
|  | P |  |  | <.0001 | 0.061 | <.0001 | <.0001 |
| Parent non-directiveness | Correlation |  |  |  | 0.15 | 0.46 | 0.16 |
|  | P |  |  |  | 0.1495 | <.0001 | 0.1207 |
| Infant liveliness | Correlation |  |  |  |  | 0.13 | 0.14 |
|  | P |  |  |  |  | 0.21 | 0.19 |
| Mutuality | Correlation |  |  |  |  |  | 0.78 |
|  | P |  |  |  |  |  | <.0001 |

Therefore, based on these analyses, the three ERP and five PCI variables were used to address the main objective of the study. Resulting PCA factors themselves were not used to avoid complicating interpretation and to enable relating the findings to existing literature on each of these theoretically independent measures. Correlations among the selected variables are reported in Table A5.

**Table A5. Full matrix of uncorrected association between ERP response difference to dynamic gaze shifts (Toward vs. Away) and measures of parent-child interaction.**

|  |  |  | Infant positive affect | Infant attentiveness | Parent sensitive responsiveness | Infant liveliness | Mutuality |
| --- | --- | --- | --- | --- | --- | --- | --- |
| Control | Amplitude P400 | Correlation | 0,20 | 0,15 | 0,15 | 0,10 | 0,12 |
| Group |  | P | 0,19 | 0,35 | 0,32 | 0,54 | 0,43 |
|  | Latency P1 | Correlation | 0,39 | 0,05 | 0,42 | 0,17 | 0,29 |
|  |  | P | 0,01 | 0,76 | 0,00 | 0,28 | 0,06 |
|  | Latency P400 | Correlation | 0,07 | 0,22 | -0,02 | 0,10 | 0,18 |
|  |  | P | 0,64 | 0,15 | 0,91 | 0,52 | 0,26 |
| At-Risk | Amplitude P400 | Correlation | -0,19 | 0,08 | -0,07 | -0,11 | -0,06 |
| Group |  | P | 0,24 | 0,63 | 0,68 | 0,49 | 0,72 |
|  | Latency P1 | Correlation | 0,04 | 0,26 | 0,07 | -0,22 | 0,24 |
|  |  | P | 0,81 | 0,10 | 0,68 | 0,18 | 0,13 |
|  | Latency P400 | Correlation | 0,39 | 0,10 | 0,06 | -0,08 | 0,01 |
|  |  | P | 0,01 | 0,53 | 0,72 | 0,63 | 0,94 |

**Table A6-1: Slopes estimate ERP measures in control group (Model 1)**

| ERP measures | Parameters | Estimation | SE | t-test | Pr > |t| |
| --- | --- | --- | --- | --- | --- |
| P100 latency | Intercept | -9,03 | 5,89 | -1,53 | 0,13 |
|  | Sensitive responsiveness | 4,52 | 1,52 | 2,98 | 0,00 |

**Table 6-2: Slopes estimate ERP measures in at risk group (Model 3)**

| ERP measures | Parameters | Estimation | SE | t-test | Pr > |t| |
| --- | --- | --- | --- | --- | --- |
| P400 latency | Intercept | -38,67 | 15,95 | -2,42 | 0,02 |
|  | Positive affect | 10,15 | 3,81 | 2,66 | 0,01 |
